# Medical College Circulars

**Published:** 1850-10

**Authors:** 


*Medical College Circulars.*—We quote the following paragraph from our esteemed contemporary, the Western Lancet, simply stating at this time that it expresses views which have suggested themselves to us in reading not a few of the many Circulars with which we are favored. If we are not mistaken, the honor of the profession requires a reform in the tone of these annual missives. One way to bring this about is for Editors of medical journals to review those circulars which are obnoxious to criticism.

*Medical College Circulars.*—We wish to call the attention of our friends, the Professors of Medical Colleges, and also the Editors of Medical Journals, to the style of communication which has for the last few years crept into the circulars of our medical schools. It is not necessary to allude to the origin of this practice, or to designate one college more than another, for all are more or less implicated in it. It appears to us that the style of these documents might be much improved, and made to conform more to professional propriety than they do at present, with advantage. We see no reason why a body of men, collectively, ought to say that about themselves, that would be improper for any one to say about himself as an individual. Professional etiquette and propriety prohibit puffing and laudatory notices of one's self. Ought it not to be equally binding on an association of medical men. We have thrown out these few thoughts without intending to cast censure, but for reflection; if we are wrong, we do not object to being set right.

